# O-GlcNAcylation Reduces Ischemia-Reperfusion–Induced Brain Injury

**DOI:** 10.1038/s41598-017-10635-0

**Published:** 2017-09-06

**Authors:** Jin-hua Gu, Jianhua Shi, Chun-ling Dai, Jian-bin Ge, Yang Zhao, Yanxing Chen, Qian Yu, Zheng-hong Qin, Khalid Iqbal, Fei Liu, Cheng-Xin Gong

**Affiliations:** 1 Jiangsu Key Laboratory of Neuroregeneration, Co-Innovation Center of Neuroregeneration, Nantong University, Nantong, Jiangsu 226001 China; 2 Department of Neurochemistry, Inge Grundke-Iqbal Research Floor, New York State Institute for Basic Research in Developmental Disabilities, Staten Island, New York, 10314 USA; 3 Department of Pathophysiology, Nantong University, Nantong, Jiangsu 226001 China; 4 Department of Pharmacology and Laboratory of Aging and Nervous Diseases, Soochow University School of Pharmaceutical Science, Suzhou, 215123 China; 5 Department of Neurology, The First Hospital of Jilin University, Xinmin Street, Changchun, Jilin 130021 China; 6 Department of Neurology, the Second Affiliated Hospital, School of Medicine, Zhejiang University, Hangzhou, 310009 China; 7 Department of Orthopedics, Qianfoshan Hospital, Shandong University, Jinan, 250014 China

## Abstract

O-GlcNAcylation is a common posttranslational modification of nucleocytoplasmic proteins with β-N-acetylglucosamine (GlcNAc) and regulates numerous biological processes. By using mouse models of cerebral ischemia induced by permanent and transient middle cerebral artery occlusion (MCAO), we observed an initial elevation (~1.7-fold, 1–4 hours after ischemia) and then decline of O-GlcNAcylation during cerebral ischemia. We found that moderate increase (<3-fold) of brain O-GlcNAcylation by pharmacological means ameliorated cerebral ischemia-reperfusion injury and the consequent motor and neurological deficits. Interference of the transient elevation of O-GlcNAcylation pharmacologically or genetically aggravates the ischemia-induced brain damage, motor deficits and mortality. The alteration of O-GlcNAcylation was also seen in the ischemic areas of postmortem human brains. This study reveals an important regulation of cerebral ischemia-reperfusion injury by O-GlcNAcylation and also provides a possible therapeutic strategy, i.e., by increasing O-GlcNAcylation, to reduce the cerebral damage and improve the clinical outcome of ischemic stroke.

## Introduction

Stroke is mostly caused by occlusion of a cerebral artery (ischemic stroke) and is a leading cause of brain injury that strikes approximately 800,000 people and kills approximately 150,000 people each year in the United States alone^1^. Cellular responses post-ischemic brain injury are critical to recovery and survival. Therefore, better understanding of the post ischemic cellular responses will help develop more effective strategies to counteract ischemic brain injury.

Protein O-GlcNAcylation is a posttranslational modification of nucleocytoplasmic proteins with O-linked β-N-acetylglucosamine (GlcNAc). It plays a critical role in regulating many biological processes^2^. O-GlcNAcylation also serves as a nutrient and stress sensor of the cell^3–5^. A range of stress stimuli cause an acute increase in protein O-GlcNAcylation. Marked changes of O-GlcNAcylation are seen in ischemic heart tissue and in diabetes^6–12^. We and others recently found a temporary elevation of protein O-GlcNAcylation in the mouse brain after ischemic brain injury^13, 14^. However, how the elevation of brain O-GlcNAcylation impacts the brain injury and repair remains to be investigated.

In the present study, we investigated the role of the transient elevation of brain O-GlcNAcylation in the injury and recovery post ischemia by using the MCAO (middle cerebral artery occlusion) mouse models. We further demonstrated the dynamic changes of brain O-GlcNAcylation both in the brains of mice after MCAO and in the ischemic human brain tissue. Most excitingly, we found that pharmacological elevation of O-GlcNAcylation ameliorates cerebral ischemia-reperfusion injury.

## Results

### Cerebral ischemia induces dynamic changes of O-GlcNAcylation

**Figure 1:**
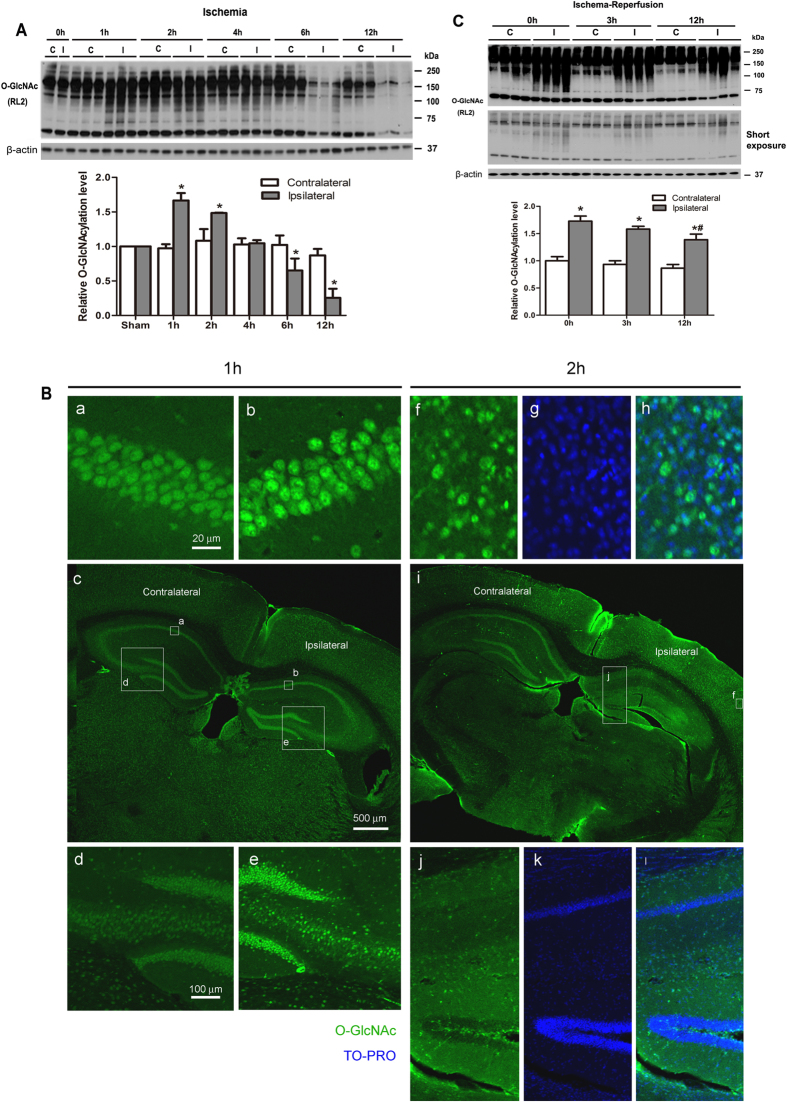
Dynamic alterations of brain O-GlcNAcylation after ischemia and ischemia-reperfusion injury. (**A**) Western blots of global O-GlcNAcylated proteins in the cerebrocortical homogenates from mice 0–12 hrs after MCAO. Quantifications of all the positive bands of the blots are shown in the graph. (**B**) Frozen coronal brain sections of mice sacrificed 1 (a-e) or 2 h (f-l) after MCAO at the right side were prepared and stained with a mixture of monoclonal antibodies RL2 and CTD110.6 for O-GlcNAc (green) and with TO-PRO (blue, pseudo color) for nuclear staining. The squares and rectangles labeled with small case letters in panels c and i indicate the areas shown in the enlarged panels labeled with the same letters. (**C**) Western blots of global O-GlcNAcylated proteins in the cerebrocortical homogenates from mice after MCAO for 2 hrs, followed by reperfusion for the indicated times (0, 3 or 12 hrs). Quantifications of all the positive bands of the blots are shown in the graph. β-Actin blots were included as loading controls for western blots. Data are presented as Mean ± SEM (n = 8/group). *p < 0.05 vs. contralateral; ^#^p < 0.05 vs. 0 h ipsilateral group. S, sham; C, contralateral; I, ipsilateral.

Because most brain alterations post ischemia are dynamic, we determined protein O-GlcNAcylation levels in the mouse brain after MCAO model for various periods of time by using Western blots developed with monoclonal antibody RL2 that recognizes global O-GlcNAcylated proteins (Table 1). We found that MCAO induced a transient increase (~1.7-fold) in protein O-GlcNAcylation in the ipsilateral cerebral cortex of the mouse brains during 1–2 hrs after cerebral artery occlusion (Fig. 1A). After 2 hrs post occlusion, the O-GlcNAcylation level started to reduce to less than 30% of the level of the contralateral side by 12 hrs. There was no significant change in the O-GlcNAcylation level in the contralateral side of the mouse brain except a slight reduction at 12 hrs after MCAO. These results indicate a transient elevation followed by a marked reduction in brain O-GlcNAcylation in the ischemic brain tissue. Such a dynamic elevation of protein O-GlcNAcylation in the mouse brains 1–2 hrs post MCAO was also seen by using double immunofluorescence staining of the brain tissue sections. A marked increase in neuronal O-GlcNAc staining (by using a mixture of antibodies RL2 and CTD110.6 to maximize the recognition of O-GlcNAc-modified proteins) was seen in the affected brain regions 1–2 hrs post MCAO (Fig. 1B). The O-GlcNAc staining was most intense in the neuronal nuclei and also in the cytoplasmic compartment of neurons (Fig. 1B, see double immunofluorescence staining in panels f-h and j-l), which is consistent with the nucleocytoplasmic localization of the O-GlcNAcylation modification in the brain^15^.

**Table 1:** Primary antibodies used in this study.

Antibody	Type	Specificity	Source/Reference
RL2	Mono-	O-GlcNAc	Thermo Scientific
CTD110.6	Mono-	O-GlcNAc	Covance, Emeryville, CA
GLUT1	Poly-	GLUT1	Millipore, Temecula, CA
GLUT2	Poly-	GLUT2	Milipore, Billerica, MA
GLUT3	Poly-	GLUT3	Santa Cruz Biotechnology, CA
GFAT2(H-300)	Poly-	GFAT2	Santa Cruz Biotechnology
OGA	Poly-	OGA	Crawford *et al*., 2008^37^
OGT	Poly-	OGT	Sigma, St. Lious, MO
β-actin	Mono-	β-actin	Sigma

Reperfusion after cerebral ischemia often induces further tissue damage and is critical to the final outcome of stroke^16^. We therefore investigated protein O-GlcNAcylation during reperfusion after MCAO for two hrs. We found that the elevated brain O-GlcNAcylation lasted for a much longer time during reperfusion as compared with permanent cerebral ischemia. Although the O-GlcNAcylation level slightly decreased during the first 12 hrs of reperfusion, it was still much higher in the ipsilateral side than the contralateral side (Fig. 1C) up to 12 hrs after reperfusion. Therefore, while brain O-GlcNAcylation is increased only transiently for 1–2 hrs after ischemia, this increase lasts many hours after reperfusion.

### O-GlcNAcylation ameliorates cerebral ischemia-reperfusion injury

**Figure 2:**
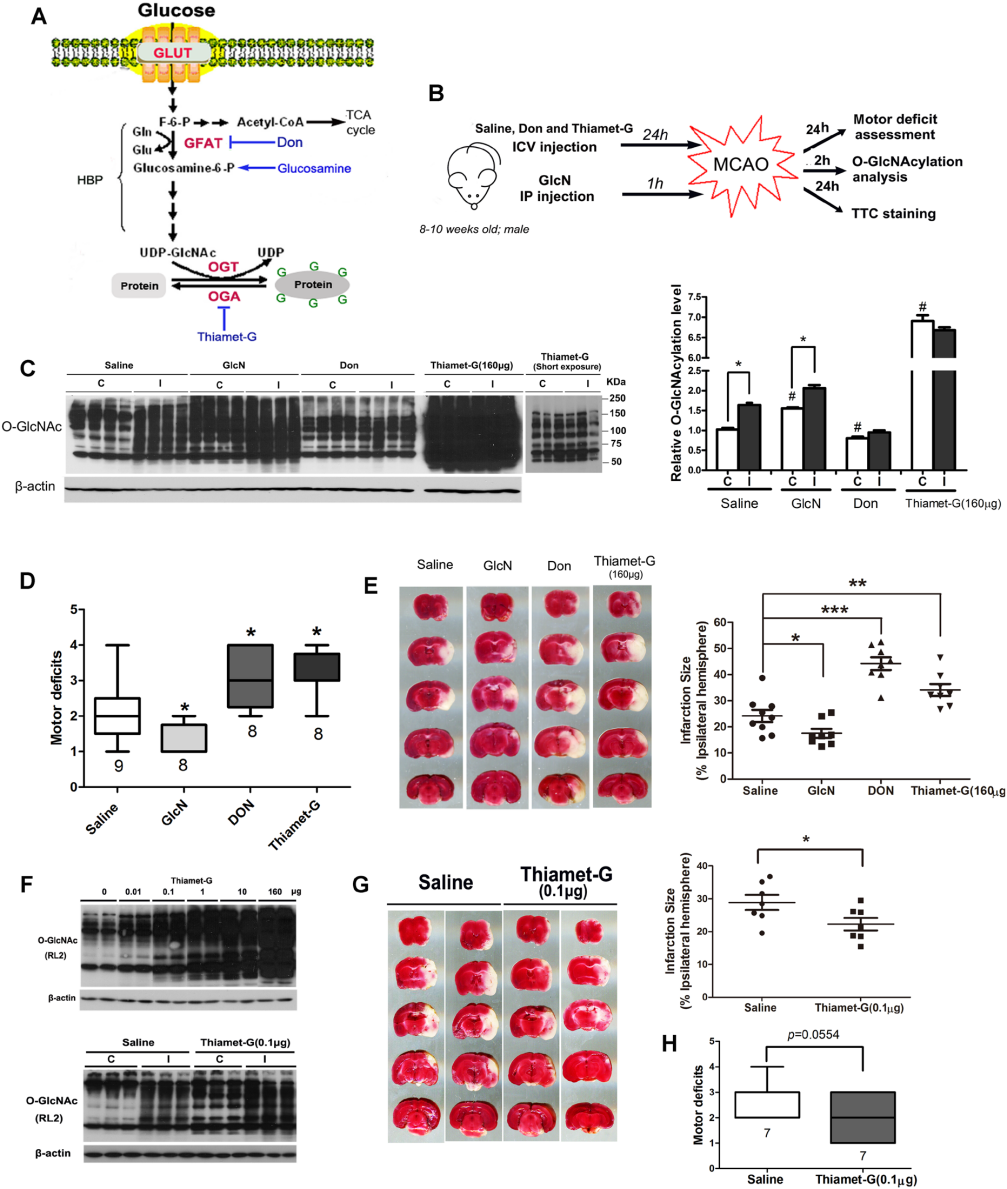
HBP and O-GlcNAcylation pathway and modulation of ischemia-reperfusion injury by O-GlcNAcylation. (**A**) Diagram of HBP and O-GlcNAcylation pathway (left panel). The pharmacological manipulations used in this study are indicated in blue color. (**B**) Schematic presentation of the experimental design. GlcN, Glucosamine. (**C**) Western blots for O-GlcNAcylation of striatum homogenates from mice 2 hrs after MCAO. Quantification of all the positive bands of the blots is shown as mean ± SEM (n = 8–9/group) in the graph. *p < 0.05 vs. contralateral; ^#^p < 0.05 vs. saline group. (**D**) Scores of motor deficits (median ± 40%) detected after MCAO for 2 hrs and reperfusion for 24 hr. The numbers under the columns indicate the number of mice used. *p < 0.05 vs. saline group. **(E**) TTC staining of the brain slices from mice after MCAO for 2 hrs followed by reperfusion for 24 hrs. Quantification of the infarct sizes detected by TTC staining is shown in the graph. *p < 0.05 vs. saline group. (**F**) Western blots for O-GlcNAcylation of striatum homogenates from mice 2 hrs after MCAO. The mice received icv injection of the indicated amounts of thiamet-G 24 hrs before MCAO. (**G**) TTC staining of the brain slices from mice after MCAO for 2 hrs followed by reperfusion for 24 hrs. The quantifications are shown as mean ± SEM (n = 8/group) in the graph at the right side (*p < 0.05 vs. saline group). (**H**) Scores of motor deficits (median ± 40%) detected after MCAO for 2 hrs and reperfusion for 24 hr. The numbers under the columns indicate the number of mice used for the group.

Protein O-GlcNAcylation is catalyzed by O-GlcNAc transferase (OGT), and the O-GlcNAc of proteins can be removed by O-GlcNAcase (OGA)^2^ (Fig. 2A). Protein O-GlcNAcylation is also regulated by the intracellular level of UDP-GlcNAc that, as the donor substrate for OGT, provides GlcNAc for protein O-GlcNAc modification. UDP-GlcNAc is synthesized in the cells from glucose through the hexosamine biosynthetic pathway (HBP), of which glutamine:fructose-6-phosphate amidotransferase (GFAT2) is the rate-limiting enzyme of in the brain^17^. To investigate whether the alteration of O-GlcNAcylation affects cerebral ischemia-reperfusion injury and recovery, we manipulated the brain O-GlcNAcylation level pharmacologically and then studied its impact on motor function of the mice and the infarct size (Fig. 2B). We induced reduction of brain O-GlcNAcylation by treating mice with 2.6 μg/mouse 6-diazo-5-oxonorleucine (DON) (a GFAT inhibitor)^18^ and elevation of brain O-GlcNAcylation with 10 mg/mouse glucosamine (GlcN, which increase the HBP flux bypassing GFAT)^19^ or 160 μg/mouse thiamet-G (an OGA inhibitor)^20^ (see Fig. 2A). Western blots of brain samples collected 2 hrs after ischemic insult showed the expected changes in O-GlcNAcylation of brain proteins (Fig. 2C). We found that elevation of O-GlcNAcylation prior to ischemia with glucosamine reduced both the motor deficits (Fig. 2D) and the infarct size (Fig. 2E), as determined 24 hrs after ischemia-reperfusion. In contrast, reduction of O-GlcNAcylation prior to ischemia with DON increased both the motor deficits and the infarct size. However, a very dramatic increase in O-GlcNAcylation (>6-fold) with thiamet-G did not reduce ischemia-induced motor deficits or infarct size (Fig. 2D,E). It worsened the motor deficits and increased infarct size instead. To elucidate whether the different and opposite effects between glucosamine and thiamet-G, both of which led to elevated O-GlcNAcylation, on ischemia-induced brain injury are a drug-specific phenomenon or due to the huge difference in the O-GlcNAcylation levels produced, we titrated thiamet-G concentrations in order to find a concentration that produce a similar level of O-GlcNAcylation as with the glucosamine treatment. We found that 0.1 μg/mouse thiamet-G (icv injection) produced a comparable elevation of O-GlcNAcylation as did with 10 mg glucosamine/mouse (i.p. injection) (Fig. 2F). By using this dose of thiamet-G, we found that it reduced infarct size (Fig. 2G) and probably motor deficits too (Fig. 2H). These results suggest that a moderate elevation of O-GlcNAcylation (<3-fold increase) is neuroprotective, whereas excessive elevation of O-GlcNAcylation (>6-fold) may further damage ischemia-reperfusion-induced brain injury.

### O-GlcNAcylation improves the long-term outcome of cerebral ischemia-reperfusion injury

**Figure 3:**
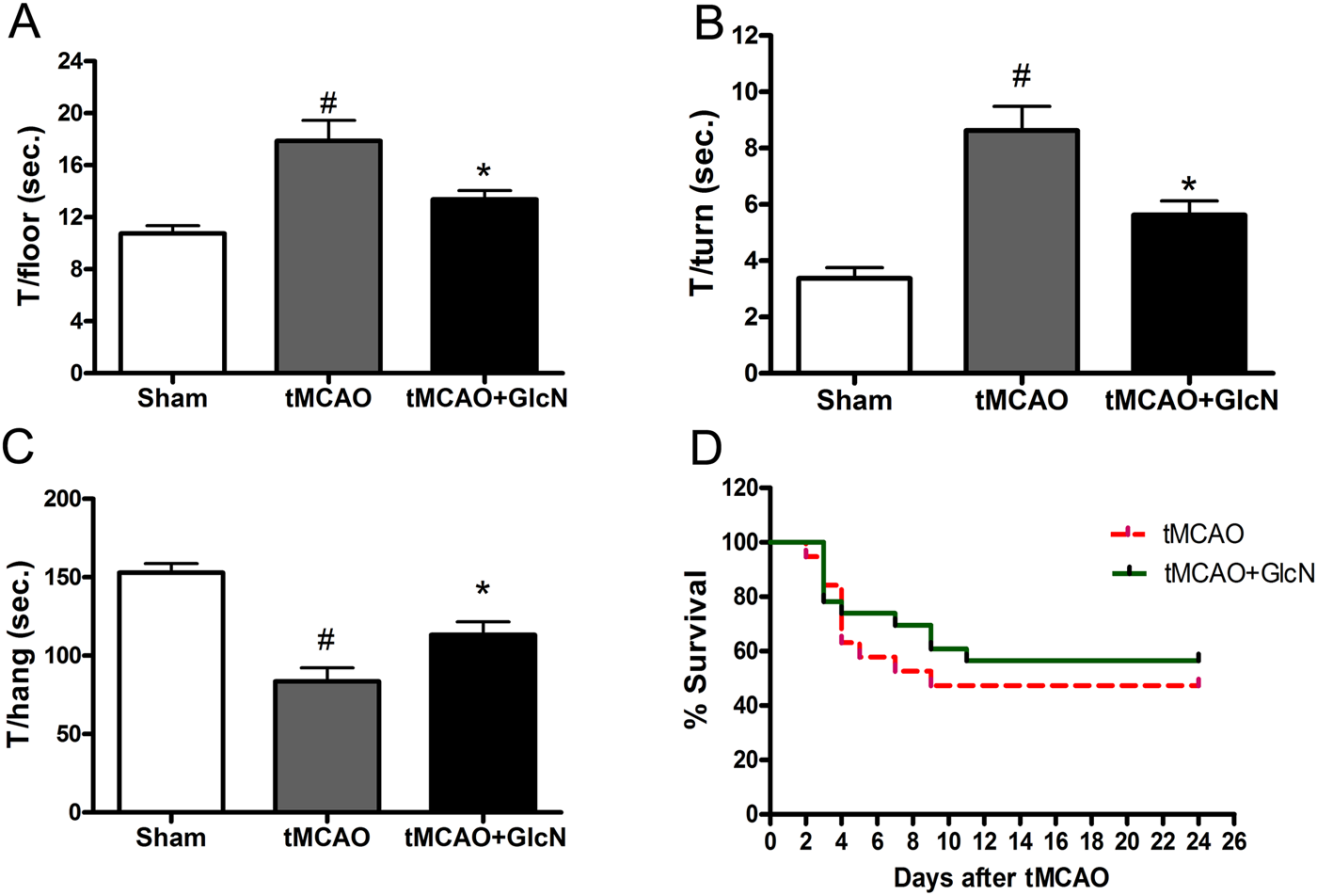
Long-term effect of glucosamine treatment on cerebral ischemia-reperfusion injury and recovery. Mice received intraperitoneal injection of glucosamine (10 mg/mouse) or vehicle, as a control, 1 hr before transient MCAO and then daily for the subsequent 24 days. The neurological and motor function of the mice were assessed using pole test and wire-hanging test during day 21–24 after MCAO/reperfusion. The time the mice took to reach the floor (T/floor) and to turn the head downwards (T/turn) in the pole test (**A**,**B**) and the time the mice stayed hanging on the wire (T/hang) in the wire-hanging test (**C**) are presented. #p < 0.05 vs. sham group; *p < 0.05 vs. tMCAO group (n = 10 in sham group; n = 9 in tMCAO group; n = 11 in tMCAO + GlcN group). The survival rates of the mice are presented in (**D**) (p = 0.16 between the two groups by log-rank test).

To evaluate the long-term effect of the upregulation of O-GlcNAcylation on cerebral ischemia-reperfusion injury and recovery, we examined the motor function of mice three weeks after the ischemia-reperfusion injury with or without glucosamine treatment (10 mg/mouse/day, i.p. injection) that increased brain O-GlcNAcylation level (see Fig. 2B). We found that glucosamine treatment significantly improved the motor function, as assessed by pole test (Fig. 3A and B) and wire-hanging test (Fig. 3C). The treatment appeared to improve the post ischemic survival rate as well, though the differences between the two survival curves did not reach statistical significance (Fig. 3D). Thus, upregulation of O-GlcNAcylation can improve the long-term outcome of cerebral ischemia-reperfusion injury.

### Neuronal OGT KO aggravates cerebral ischemia-reperfusion injury

**Figure 4:**
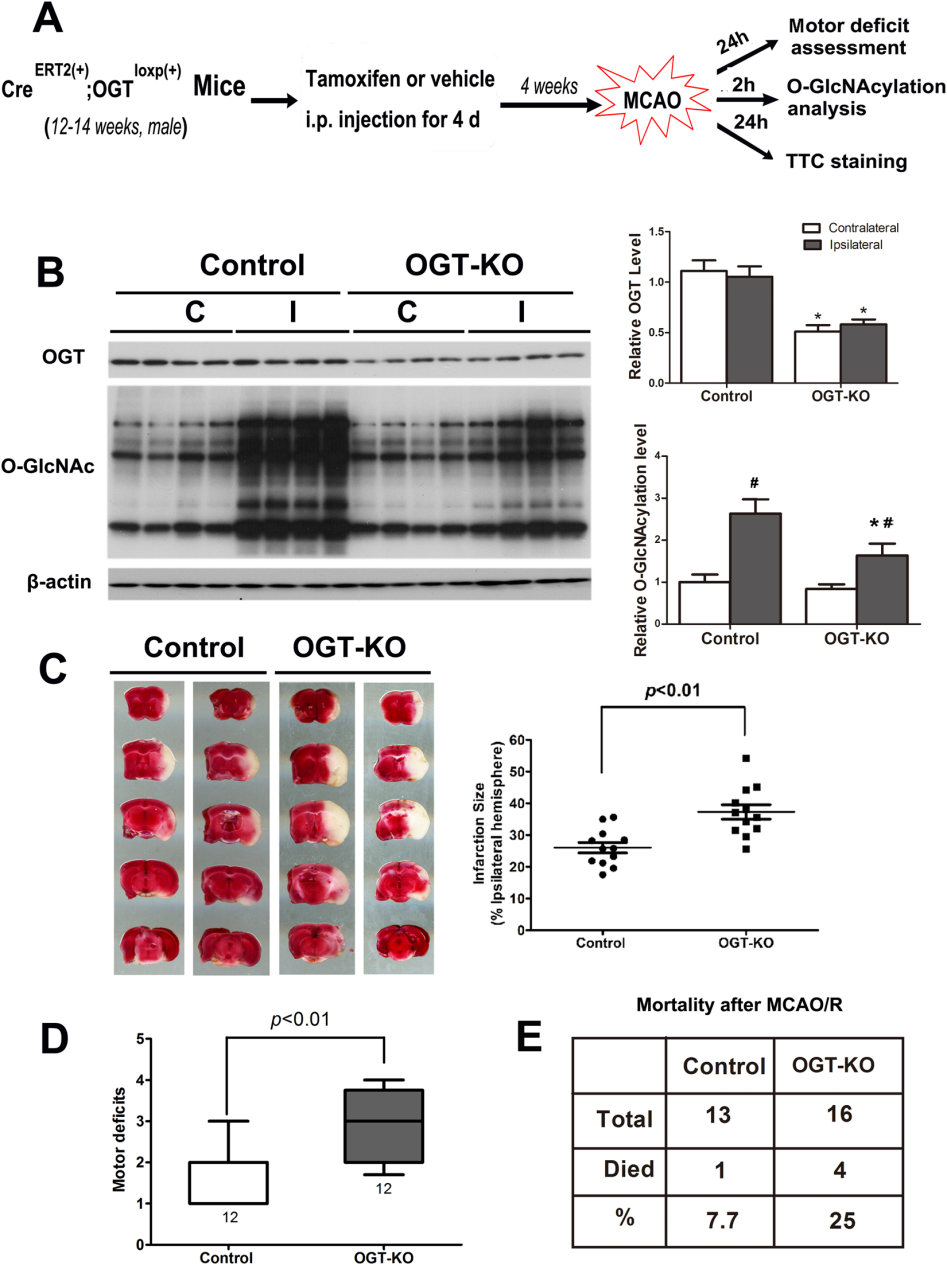
Infarct size, motor deficit and mortality after ischemia-reperfusion injury in neuronal OGT KO mice. (**A**) Schematic presentation of the experimental design. (**B**) Western blots for the levels of OGT and O-GlcNAcylation in the striatum of mice 2 hrs after MCAO. Quantification of the blots (mean ± SEM, n = 8/group) is shown in the graphs. *p < 0.05 vs. control group; ^#^p < 0.05 vs. contralateral group. (**C**) TTC staining of the brain slices from mice after MCAO for 2 hrs followed by reperfusion for 24 hrs. Quantification (mean ± SEM) of the infarct sizes detected by TTC staining is shown in the graph. (**D**) Scores of motor deficits (median ± 40%) of mice after MCAO/reperfusion for 24 hrs. The numbers under the columns indicate the number of mice. (**E**) Mortality of mice after MCAO.

To confirm the role of neuronal O-GlcNAcylation on cerebral ischemia-reperfusion injury, we developed a neuron-specific brain OGT knockout (KO) mouse model, in which the OGT gene was floxed by two loxP sites and the CRE expression was induced by tamoxifen administration and controlled by the CaMK-IIα promoter. The mice were subjected to MCAO four weeks after induction of neuronal OGT KO with tamoxifen injection for consecutive four days. We then determined the OGT and O-GlcNAcylation levels in the striatum (the major ischemia site after MCAO) by Western blots two hours after ischemia, as well as assessed motor deficits and detected brain tissue damage 24 hrs after reperfusion in mice (Fig. 4A). As expected, we observed approximately 50% reduction in the level of OGT in the striatum of the OGT KO mice as compared to the mice without KO induction (Fig. 4B), confirming the success of inducing neuronal OGT KO four weeks after tamoxifen injection. The remaining OGT was likely from non-neuronal cells of the brain tissue. In the ischemic brain tissue, the protein O-GlcNAcylation was markedly increased two hours after MCAO in the control mice, but this increase was partially prevented in the neuronal OGT KO mice (Fig. 4B). We found that when the elevation of O-GlcNAcylation was partially diminished, the MCAO-induced brain infarct size was larger (Fig. 4C) and the motor deficits were more severe (Fig. 4D) in the neuronal OGT KO mice than in control mice. MCAO also produced more mortality in the neuronal OGT KO mice (25%) than in the control mice (7.7%) (Fig. 4E). These results suggest that prevention of the dynamic elevation of neuronal O-GlcNAcylation aggravates cerebral ischemia-reperfusion injury and thus further support the protective role of O-GlcNAcylation during early ischemia-reperfusion injury.

### Protein O-GlcNAcylation level alters in ischemic human brain tissue

**Figure 5:**
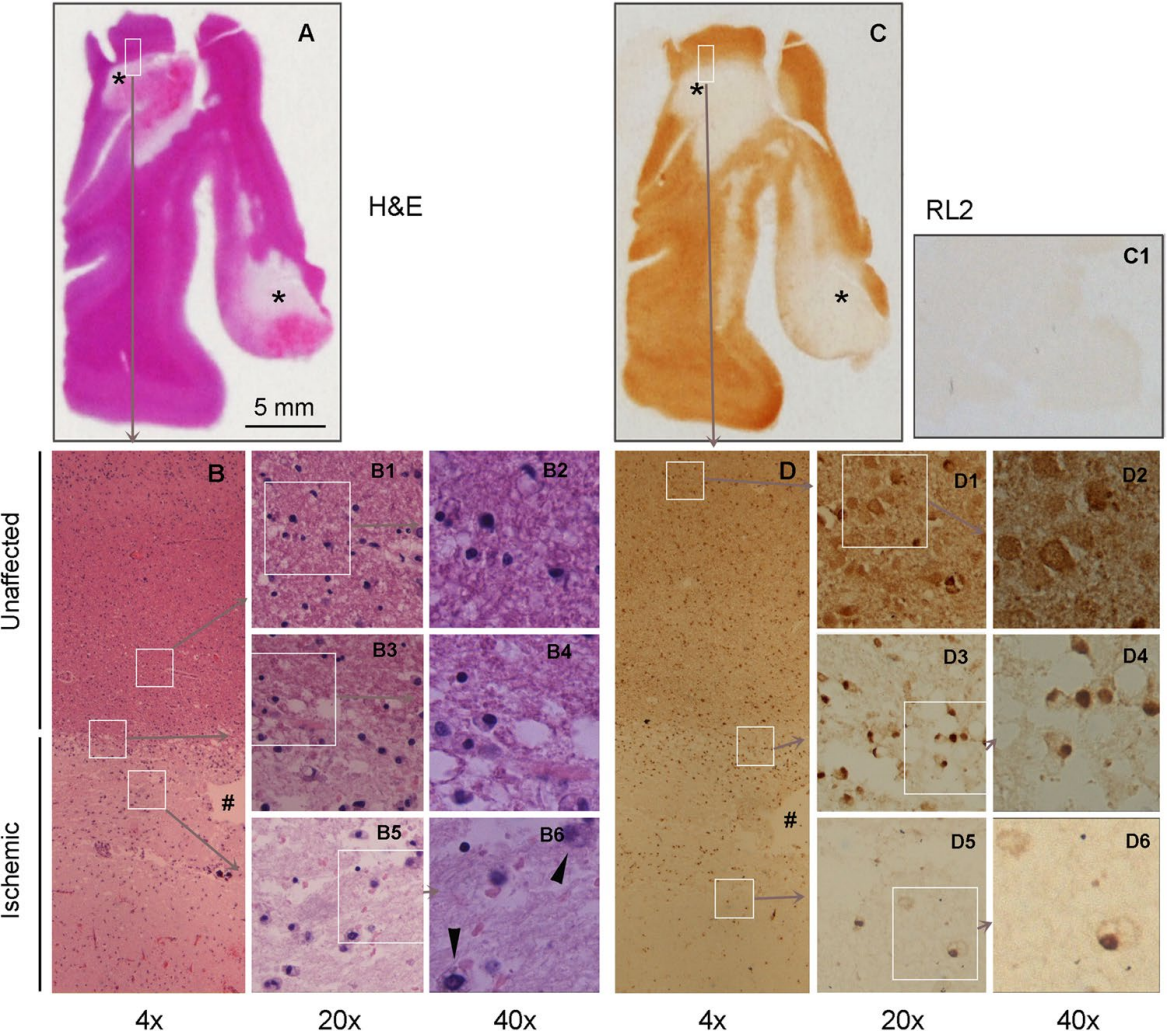
Histology and O-GlcNAc immunohistochemistry of ischemic human brain tissue. Adjacent paraffin sections of the middle frontal gyrus of the postmortem brain from an 82-yesr-old, female individual were stained with hematoxylin and eosin (H&E) (**A**, **B**) or monoclonal antibody RL2 against O-GlcNAcylated proteins (**C**, **D**). Control staining was carried out when RL2 was eliminated from the primary antibody solution (C1). Asterisks in panels A and C indicate the ischemic area. White rectangles and squares indicate the areas for images of higher magnifications. ^#^Indicates the tissue marker used for identification of the same areas in the adjacent sections. Arrowheads in panel B6 indicate macrophages.

To learn whether protein O-GlcNAcylation is altered in the human brain after ischemic stroke, we stained the adjacent paraffin sections of four postmortem human brains, which all contained both the ischemic and unaffected areas of brain tissue. The ischemic area of brain tissue was confirmed by histological examination of the tissue sections stained with hematoxylin and eosin (H&E). It is well established that the ischemic brain tissue has much paler H&E staining than the unaffected normal brain tissue. We used this approach to localize the ischemic brain area (Fig. 5A and B), which was confirmed by the loss of neuronal cells as well as the appearance of macrophages and vacuolization (Fig. 5B3-B6). Comparison between the H&E staining (Fig. 5A) and O-GlcNAc staining (Fig. 5C) of the adjacent section indicated marked weaker immunostaining of O-GlcNAc (stained with monoclonal antibody RL2 against O-GlcNAcylated proteins) in the ischemic areas as marked by asterisks. The RL2 staining was specific because its omission from the primary antibody solution, as a control, eliminated the staining (Fig. 5C1). Under higher magnifications, O-GlcNAc was seen both in the nuclei and cell bodies and neurites (Fig. 5D1 and D2) in the unaffected cerebral cortex, which is consistent with our previous observations^15^. However, at the border region between the normal and the ischemic core cortex (penumbra), the O-GlcNAc staining was found to be condensed in the nuclei with marked decrease or loss of the O-GlcNAc staining in the cytoplasmic compartment (Fig. 5D3 and D4). Vacuolization lesion was also obvious with O-GlcNAc staining at the border region. The O-GlcNAc staining was almost lost in the ischemic regions, leaving positive staining in the nuclei of some macrophages (Fig. 5D 5 and D6). These results indicate a marked decrease of O-GlcNAcylation after ischemia in the human brain. The marked reduction of O-GlcNAcylation observed in the ischemic human brain tissue is consistent with the marked reduction of O-GlcNAcylation at the second phase of ischemic mouse brains because stroke did not occur immediately before death in these human cases.

## Discussion

In the present study, by using the MCAO mouse model, we demonstrated an initial elevation (1–4 hours after ischemia) and then marked decline of protein O-GlcNAcylation during permanent cerebral ischemia. If reperfusion occurred two hours after ischemia, the elevation of O-GlcNAcylation lasted longer (up to at least 12 hrs after reperfusion, the longest time point in this study). Additionally, we discovered, for the first time, changes of protein O-GlcNAcylation in ischemic human brain tissue. Most importantly, we found through modulation of brain O-GlcNAcylation that the elevation of brain O-GlcNAcylation is neuroprotective and that pharmacological elevation of brain O-GlcNAcylation ameliorates cerebral ischemia-reperfusion injury.

O-GlcNAcylation is a known sensor of intracellular glucose metabolism, and decreased intracellular glucose usually results in decreased O-GlcNAcylation because the donor for O-GlcNAcylation, UDP-GlcNAc, is generated from glucose metabolism through the HBP. However, protein O-GlcNAcylation also responds to various stresses^21^. Transient elevation of O-GlcNAcylation is known to occur under conditions of decreased intracellular glucose concentration. A transient elevation of O-GlcNAcylation was reported after glucose deprivation in cultured HepG2 cells^22^, Neuro-2a neuroblastoma cells^23^, and cardiomyocytes^24^. Interestingly, the mechanisms leading to the transient elevation of O-GlcNAcylation appear to be different among various cell types and/or conditions. In liver Hep2G cells, glucose deprivation may stimulate O-GlcNAcylation through up-regulation of OGT and concomitant decrease in OGA levels^22^. In Neuro-2a cells, glucose deprivation caused only up-regulation of OGT expression. In cardiomyocytes, the glucose deprivation–induced increase in O-GlcNAcylation was calcium-dependent^24^. The dynamic alterations of brain O-GlcNAcylation post ischemia in mammalian brain appears to be X-box-binding protein-1−dependent^25^, but the exact molecular mechanisms remains elusive.

The observed increase in brain O-GlcNAcylation during early ischemia may represent a stress response aimed at preserving protein structure and function, although it can also promote apoptosis^13^. Our finding that O-GlcNAcylation is neuroprotective to the ischemia-reperfusion injury is consistent to this notion. Hart and colleagues have reported that a wide variety of stress stimuli rapidly increase O-GlcNAcylation. Inhibition of this post-translational modification makes cells more vulnerable to stress insults and results in decreased survival of cultured cells^26, 27^. In contrast, increasing O-GlcNAc levels protects cells. In heart ischemia, increased O-GlcNAcylation is protective, preventing tissue damage in infarcted heart^28^, whereas cardiomyocyte OGT knockout exacerbates ischemia-induced cardiac dysfunction^29^. However, if O-GlcNAcylation is elevated chronically, as it occurs in diabetes, the sugar modification may contribute directly to cardiomyopathy^6, 12^. Furthermore, the neuroprotection by the transient elevation of brain O-GlcNAcylation might disappear during aging because a recent study reported that, unlike young mice, aged mice fail to increase O-GlcNAcylation in response to transient cerebral ischemia^14^. A recent study reported that pharmacological elevation of O-GlcNAcylation before or after stroke improves the outcome in old mice as well^25^.

To explore the role of the dynamic elevation of O-GlcNAcylation in cerebral ischemia, we used three pharmacological compounds (DON, glucosamine and thiamet-G) to manipulate the level of O-GlcNAcylation in the present study. We observed a moderate elevation and reduction of brain O-GlcNAcylation with glucosamine and DON, respectively. The moderate change of O-GlcNAcylation with these two compounds ameliorated and aggravated, respectively, ischemia-induced brain damage and motor deficits in mice, suggesting a neuroprotective role of O-GlcNAcylation during cerebral ischemia-reperfusion injury. Our findings are consistent with previous reports showing that GlcN can reduce ischemia/reperfusion-induced cerebral infarct size^19^ and retinal thinning^30^ in rats. However, our initial dose of thiamet-G (160 μg/mouse), which was selected according to the dose used previously^31^, led to an excessive (>6-fold) elevation of O-GlcNAcylation and more severe brain damage and motor deficits after MCAO in mice. When we lowered the thiamet-G dose to that resulting in a moderate elevation of O-GlcNAcylation, we again observed a neuroprotective role against cerebral ischemia insult. During the preparation of this manuscript, two studies reported that intraperitoneal administration of thiamet-G at a dose of 20 or 30 mg/kg, which also led to a moderate increase in brain protein O-GlcNAcylation, rendered neuroprotection against experimental stroke^25, 32^. Taken together, it appears that the transient, moderate elevation of O-GlcNAcylation during the early phase of cerebral ischemia is neuroprotective, but the excessive elevation of O-GlcNAcylation is detrimental. This double-edged sword phenomenon is actually common in other type of responses to stress and insults, such as neuroinflammation and apoptosis^27^.

To verify the role of O-GlcNAcylation in cerebral ischemia-reperfusion injury, we studied the MCAO-induced brain damage and motor deficits in mice after the neuronal OGT was selectively knocked out. We found that the ischemia-induced transient elevation of O-GlcNAcylation was markedly prevented and, consequently, the brain damage and motor deficits were significantly more severe in these mice. A very marked increase in the mortality was also observed in the OGT KO mice after MCAO. These results further support the neuroprotective role of O-GlcNAcylation during cerebral ischemic injury. It is worth noting that OGT KO in glial cells might have a similar or different impact on ischemic brain injury. Future studies using OGT KO mice restricted in glial cells, which are not currently available, will help reveal the role of glial OGT or glial O-GlcNAcylation in ischemic brain injury.

The dynamic alteration of O-GlcNAcylation during cerebral ischemia-reperfusion injury that we observed in the mouse brain likely occurs in the human brain too. Though it is not possible to determine the time-course changes of O-GlcNAcylation in human ischemic brain tissue, we studied the O-GlcNAcylation level of postmortem human brain tissue sections that contained both ischemic and unaffected tissue from individuals with ischemic stroke. We found marked reduction and even loss of the O-GlcNAc staining in the ischemic regions of the human brain tissue sections, which is consistent to the later stages (after six hours post ischemia) of cerebral ischemia observed in the mouse brain. Indeed, the brain tissue samples we studied were not from the individuals killed by stroke. These brain samples were obtained from the brains of individuals who died of causes other than stroke but who had previous stroke histories. The small ischemic strokes we studied were extremely unlikely to be fresh stroke that occurred within four hours before the death. According to our observations in the mouse brains after MCAO, we expect to see increased O-GlcNAcylation in the ischemic human brain tissue if such human tissue samples within four hours after stroke were available for the study.

The mechanism underlying the role of O-GlcNAcylation in attenuating cerebral ischemia/reperfusion injury requires further investigation. We have shown that O-GlcNAcylation induces neuronal apoptosis in the MCAO mouse model^13^. Apoptosis is a double-edged sword in cerebral ischemia. Early apoptosis in cerebral ischemia will limit inflammatory and immune responses, which are detrimental to penumbra cell, causing ischemic core expansion. O-GlcNAcylation can also directly regulate inflammatory response. Glucosamine, which can increase the protein O-GlcNAcylation, render the protective effect in a MCAO model by inhibiting inflammation^19^. A recent study showed that thiamet-G, which can elevate protein O-GlcNAcylation level, may play a neuroprotective role against experimental stroke by modulating microglia/macrophage polarization and inhibiting NF-kB p65 signaling^32^. Another new study suggested that the O-GlcNAcylation-mediated neuroprotection is dependent on X-box-binding protein-1^25^.

In conclusion, we have demonstrated a dynamic alteration of O-GlcNAcylation (first elevation and then decline) during cerebral ischemia and ischemia-reperfusion injury in mice. We have shown that O-GlcNAcylation was not merely a response to cerebral ischemia; it served as a novel regulation of cerebral ischemia-reperfusion injury. The initial and transient elevation of brain O-GlcNAcylation was neuroprotective and helped ameliorate cerebral ischemia-reperfusion injury. Moderate pharmacological increase of brain O-GlcNAcylation reduced brain injury and motor deficits. These findings suggest a novel therapeutic strategy for ischemic stroke through pharmacological elevation of brain O-GlcNAcylation.

## Materials and Methods

### Reagents and antibodies

Primary antibodies used in this study are listed in Table 1. Peroxidase-conjugated anti-mouse and anti-rabbit IgG were obtained from Jackson ImmunoResearch Laboratories (West Grove, PA). Alexa 488-conjugated goat anti-mouse IgG and TO-PRO-3 iodide (642/661) were from Life Technologies (Grand Island, NY). The enhanced chemiluminescence (ECL) kit was from Thermo Scientific (Rockford, IL). Other chemicals were from Sigma (St. Louis, MO).

### Human brain tissue

Paraffin-embedded human brain tissue sections with no identifiable subject information were obtained from the Banner Sun Health Research Institute Brain and Body Donation Program (Sun City, AZ) through a fee-for-service basis under a formal material transfer agreement. The autopsy and the brain tissue collection and processing at the Banner Sun Health Research Institute was approved by its institutional review board and in accordance with the National Institutes of Health guidelines (https://www.brainandbodydonationregistration.org/). The use of the tissue sections was also approved by the institutional review board of New York State Institute for Basic Research in Developmental Disabilities (Staten Island, NY). The tissue sections used in the present study were from the non-demented control cases (with no identifiable information about the donors) for Alzheimer’s disease, and cerebral ischemia in these sections was confirmed histologically.

### Animals

All strains of mice were initially purchased from the Jackson Laboratory (New Harbor, ME) and bred in our animal facility. Mice were housed (4–5 animals per cage) with a 12/12-hr light/dark cycle and with ad libitum access to food and water. The housing, breeding, and animal experiments were approved by the Institutional Animal Care and Use Committee of Nantong University and of New York State Institute for Basic Research in Developmental Disabilities. The animal experiments were in accordance with the PHS Policy on Human Care and Use of Laboratory animals (revised March 15, 2010) and with the ARRIVE guidelines. Unless specified (see below), male C57BL/6 J mice at the age of 2–3 months were used for this study.

The Camk2aCre^(+)^-Ogt^loxp(+)/loxp(+)^ mice were generated by crossing the hemizygous B6.129S6-Tg (Camk2a-cre/ERT2)1Aibs/J Cre-expressing mice with the B6.129-Ogttm1Gwh/J OGT-floxed mice, both of which were purchased from the Jackson Laboratory and back crossed for at least 10 generations in our animal colony. The male neuronal OGT KO in the Camk2aCre^(+)^-Ogt^loxp(+)/loxp(+)^ mice was induced by intraperitoneal injection (i.p.) of tamoxifen (75 mg/kg/day for 4 consecutive days) or, as a control, vehicle (corn oil containing 10% ethanol). The mice were subjected to MCAO or sham surgery 4 weeks after tamoxifen injection.

Mice were assigned randomly to different treatment groups according to an online randomization tool (randomizer.org). To implement allocation concealment, different groups were indicated with irrelevant codes. Mice that died within 6 hrs after MCAO procedure or lost more than 20% of their body weight within 48 hours were excluded (10.9%) from the study in the long-term outcome experiment. Evaluation of neurological deficits and brain infarct size was performed by an investigator blinded to the experimental treatments. The number of mice per group used in the present study are stated in the figure legends. It was designed in reference to similar previous studies or on the basis of power analysis giving 80% power to detect a difference of 1.0 × SD between groups, using a significance criterion (α) of 0.05.

### MCAO and reperfusion

The MCAO surgery was performed as described previously^33^. Briefly, mice were anesthetized with intraperitoneal injection of 2.5% avertin (2,2,2-tribromoethanol, Sigma-Aldrich, 400 mg/kg body weight), a commonly used anesthetic for mice which produces a wide anesthetic window. Through a ventral midline incision, the right common carotid artery, internal carotid artery and external carotid artery were surgically exposed. A 6–0 nylon suture with silicon coating (Doccol Corporation, Redlands, CA) was inserted into the internal carotid artery through the external carotid artery stump and was gently advanced to occlude the middle cerebral artery. For reperfusion, the occluding filament was withdrawn after occlusion for 2 hrs. Body temperature was maintained between 37.0 °C and 37.5 °C with a heating pad during surgery. Cerebral blood flow was monitored by Laser-Doppler flowmetry and only those mice with 90% of blood flow blockade during MCAO and 85–95% recovery of blood flow during reperfusion were used for further experiments. The Sham-operated mice underwent identical surgery but the suture was not inserted. At indicated time points post MCAO or ischemia-reperfusion, mice were sacrificed by cervical dislocation.

### Evaluation of neurological deficits

For determining neurological deficits of mice, neurological assessment was performed using a 5-point scoring system 24 hr after ischemia-reperfusion surgery, as follows: 0, normal motor function; 1, flexion of torso and contralateral forelimb when mouse was lifted by the tail; 2, circling to the contralateral side when mouse held by the tail on a flat surface but normal posture at rest; 3, leaning to the contralateral side at rest; 4, no spontaneous motor activity^34^. Mice that did not show behavioral deficits immediately after reperfusion (neurological score = 0) were excluded from the study.

The pole test was adapted from Matsuura *et al*. with minor medications^35^. Mouse was placed head upward near the top of a vertical wood pole (50 cm in length and 8 mm in diameter) with rough surface. The time taken to turn completely downwards (T/turn) and the total time to reach the floor with all four paws (T/floor) were recorded. If the mouse was unable to turn completely, the time to reach the floor was attributed to T/turn. Each animal was tested for 5 trials and the average score was taken as the final pole test score.

The balance and grip strength of mice were assessed using the wire-hanging test, as described previously^36^. The test apparatus consisted of a steel wire (1 mm in diameter) stretching between two posts 60 cm above a foam pillow. Each mouse was trained to cling to the wire with their forepaws for two days before the test. On the third day, latencies to fall was tested twice (maximum: 3 min), and the average latencies were recorded.

### Evaluation of brain infarct size

Infarct sizes in the mouse brains were determined after staining with 1% 2,3,5-triphenyltetrazolium chloride (TTC) by using the Image Pro Plus software (Media Cybernetics, Silver Spring, MD). Briefly, mouse brains were cut into 2-mm-thick coronal slices with a brain-cutting matrix (ASI Instruments, Warren, MI). After incubation in the TTC solution at 37 °C for 30 min, the brain slices were transferred into 4% paraformaldehyde in PBS. The stained sections were scanned, and the infarct size was determined using the Image J software. The presence of infarction was determined by the areas that lack TTC staining. The infarct size was expressed as a percentage area of the ipsilateral hemisphere.

### Intracerebroventricular (icv) injection

Mice were first anesthetized using 2.5% avertin, and then restrained onto a stereotaxic apparatus. The bregma coordinates used for injection were: −1.0 mm lateral, −0.3 mm posterior, and −2.5 mm below. Each mouse received a single icv injection of the indicated drug in 3.0 µl 0.9% saline or, as a control, 3.0 µl 0.9% saline alone into the right ventricle of the brain. The injection was administered slowly over a period of 5 min, and the needle was retained for another 10 min before removal.

### Western blots

Brain tissue (the entire cortex or striatum) was homogenized in pre-chilled buffer containing 50 mM Tris-HCl (pH 7.4), 50 mM GlcNAc (inhibitor of OGA), 20 μM UDP (inhibitor of OGT), 2.0 mM EGTA, 2 mM Na_3_VO_4_, 50 mM NaF, 0.5 mM AEBSF, 10 μg/ml aprotinin, 10 μg/ml leupeptin and 4 μg/ml pepstatin A. Protein concentrations of the homogenates were determined by using Pierce 660 nm Protein Assay kit (Thermo Fisher Scientific Inc.). The samples were resolved in 10% sodium dodecyl sulfate polyacrylamide gel electrophoresis (SDS-PAGE) and electro-transferred onto Immobilon-P membrane (Millipore, Bedford, MA). The blots were then probed with a primary antibody, washed, and then incubated with a corresponding hrP-conjugated secondary antibody. The immunoreactivity was visualized by using the Pierce ECL Western Blotting Substrate (Thermo scientific) and exposed to a HyBlot CL autoradiography film (Denville Scientific, Inc., Metuchen, NJ). Specific immunostaining was quantified by using the Multi Gauge software V3.0 (Fuji Photo Film Co., Ltd). The relative expression level was shown as ratio of quantification of specific blots level to beta-actin level.

### Immunohistochemistry

The mice brains were fixed with 4% PFA for 24 h at 4 °C and sectioned into coronal brain slices (40-μm thick) by freezing microtome. Brain slices were then blocked with 5% normal goat serum in TBS for 30 min, followed by incubation with a primary antibody solution in TBS containing 5% goat serum and 0.1% Triton X-100 at 4 °C overnight. After washing with TBS, the sections were incubated with Alexa 488-conjugated goat anti-mouse IgG (1:1000) plus TO-PRO in TBS containing 5% goat serum and 0.1% Triton X-100 at room temperature for 1 h. The immunostaining was analyzed by using a laser scanning confocal microscope (PCM 200, Nikon).

The paraffin-embedded human brain tissue sections (6-μm-thick) were first deparaffinized, oxidized, and washed. The nonspecific binding of the sections was blocked with 5% normal goat serum for 30–45 min, followed by incubation with the monoclonal antibody RL2 (1:200) at 4 °C overnight and then with biotinylated anti-mouse lgG. The immunostaining was developed by using avidin/biotinylated horseradish peroxidase (Santa Cruz Biotechnology) and peroxidase substrate DAB. The stained sections were finally mounted on microscope slides (Brain Research Laboratories, Newton, MA), dehydrated, and covered with coverslips.

### Statistical Analysis

All statistical analysis was done using Graphpad Prism 5 software. The Student’s *t* test was performed to compare two groups. One-way ANOVA was used to analyze multiple groups. Two-way ANOVA and subsequent Tukey tests were performed to analyze time course studies. Mann-Whitney U test was used to analyze neurological scores. Data are presented as means ± SEM or median ± 40% (for neurological scores only), and *p* < 0.05 was considered statistically significant.

## References

[1] Go AS (2013). Executive summary: heart disease and stroke statistics–2013 update: a report from the American Heart Association. Circulation.

[2] Hart GW (1997). Dynamic O-linked glycosylation of nuclear and cytoskeletal proteins. Annu Rev Biochem.

[3] Wells L, Vosseller K, Hart GW (2001). Glycosylation of nucleocytoplasmic proteins: signal transduction and O-GlcNAc. Science.

[4] Love DC, Hanover JA (2005). The hexosamine signaling pathway: deciphering the “O-GlcNAc code”. Sci STKE.

[5] Hart GW, Housley MP, Slawson C (2007). Cycling of O-linked beta-N-acetylglucosamine on nucleocytoplasmic proteins. Nature.

[6] Hu Y (2005). Adenovirus-mediated overexpression of O-GlcNAcase improves contractile function in the diabetic heart. Circ Res.

[7] Fulop N (2007). Impact of Type 2 diabetes and aging on cardiomyocyte function and O-linked N-acetylglucosamine levels in the heart. Am J Physiol Cell Physiol.

[8] Jones SP (2008). Cardioprotection by N-acetylglucosamine linkage to cellular proteins. Circulation.

[9] Ngoh GA, Watson LJ, Facundo HT, Dillmann W, Jones SP (2008). Non-canonical glycosyltransferase modulates post-hypoxic cardiac myocyte death and mitochondrial permeability transition. J Mol Cell Cardiol.

[10] Ngoh GA (2009). Unique hexosaminidase reduces metabolic survival signal and sensitizes cardiac myocytes to hypoxia/reoxygenation injury. Circ Res.

[11] Fricovsky ES (2012). Excess protein O-GlcNAcylation and the progression of diabetic cardiomyopathy. Am J Physiol Regul Integr Comp Physiol.

[12] Erickson JR (2013). Diabetic hyperglycaemia activates CaMKII and arrhythmias by O-linked glycosylation. Nature.

[13] Shi J (2015). O-GlcNAcylation regulates ischemia-induced neuronal apoptosis through AKT signaling. Scientific reports.

[14] Liu S, Sheng H, Yu Z, Paschen W, Yang W (2016). O-linked beta-N-acetylglucosamine modification of proteins is activated in post-ischemic brains of young but not aged mice: Implications for impaired functional recovery from ischemic stress. J Cereb Blood Flow Metab.

[15] Liu Y (2012). Developmental regulation of protein O-GlcNAcylation, O-GlcNAc transferase, and O-GlcNAcase in mammalian brain. PLoS One.

[16] Eltzschig HK, Eckle T (2011). Ischemia and reperfusion-from mechanism to translation. Nat Med.

[17] Oki T, Yamazaki K, Kuromitsu J, Okada M, Tanaka I (1999). cDNA cloning and mapping of a novel subtype of glutamine:fructose-6-phosphate amidotransferase (GFAT2) in human and mouse. Genomics.

[18] Liu F (2009). Reduced O-GlcNAcylation links lower brain glucose metabolism and tau pathology in Alzheimer’s disease. Brain.

[19] Hwang SY (2010). Glucosamine exerts a neuroprotective effect via suppression of inflammation in rat brain ischemia/reperfusion injury. Glia.

[20] Yuzwa SA (2008). A potent mechanism-inspired O-GlcNAcase inhibitor that blocks phosphorylation of tau *in vivo*. Nat Chem Biol.

[21] Chatham JC, Not LG, Fulop N, Marchase RB (2008). Hexosamine biosynthesis and protein O-glycosylation: the first line of defense against stress, ischemia, and trauma. Shock.

[22] Taylor RP (2008). Glucose deprivation stimulates O-GlcNAc modification of proteins through up-regulation of O-linked N-acetylglucosaminyltransferase. J Biol Chem.

[23] Cheung WD, Hart GW (2008). AMP-activated protein kinase and p38 MAPK activate O-GlcNAcylation of neuronal proteins during glucose deprivation. J Biol Chem.

[24] Zou L (2012). Glucose Deprivation-induced Increase in Protein O-GlcNAcylation in Cardiomyocytes Is Calcium-dependent. J Biol Chem.

[25] Jiang M (2017). XBP1 (X-Box-Binding Protein-1)-Dependent O-GlcNAcylation Is Neuroprotective in Ischemic Stroke in Young Mice and Its Impairment in Aged Mice Is Rescued by Thiamet-G. Stroke.

[26] Zachara NE (2004). Dynamic O-GlcNAc modification of nucleocytoplasmic proteins in response to stress. A survival response of mammalian cells. J Biol Chem.

[27] Zachara NE, Hart GW (2004). O-GlcNAc a sensor of cellular state: the role of nucleocytoplasmic glycosylation in modulating cellular function in response to nutrition and stress. Biochim Biophys Acta.

[28] Chatham JC, Marchase RB (2010). The role of protein O-linked beta-N-acetylglucosamine in mediating cardiac stress responses. Biochim Biophys Acta.

[29] Watson LJ (2010). O-linked beta-N-acetylglucosamine transferase is indispensable in the failing heart. Proc Natl Acad Sci USA.

[30] Chen YJ (2015). Protective effects of glucosamine on oxidative-stress and ischemia/reperfusion-induced retinal injury. Investigative ophthalmology & visual science.

[31] Yu Y (2012). Differential effects of an O-GlcNAcase inhibitor on tau phosphorylation. PLoS One.

[32] He, Y., Ma, X., Li, D. & Hao, J. Thiamet G mediates neuroprotection in experimental stroke by modulating microglia/macrophage polarization and inhibiting NF-kappaB p65 signaling. *J Cereb Blood Flow Metab*, doi:10.1177/0271678X16679671 (2016). 10.1177/0271678X16679671 PMC5536801 27864466

[33] Clark WM, Lessov NS, Dixon MP, Eckenstein F (1997). Monofilament intraluminal middle cerebral artery occlusion in the mouse. Neurol Res.

[34] Bederson JB (1986). Rat middle cerebral artery occlusion: evaluation of the model and development of a neurologic examination. Stroke.

[35] Matsuura K, Kabuto H, Makino H, Ogawa N (1997). Pole test is a useful method for evaluating the mouse movement disorder caused by striatal dopamine depletion. Journal of neuroscience methods.

[36] Gertz K (2006). Physical activity improves long-term stroke outcome via endothelial nitric oxide synthase-dependent augmentation of neovascularization and cerebral blood flow. Circ Res.

[37] Crawford GL, Hart GW, Whiteheart SW (2008). Murine platelets are not regulated by O-linked beta-N-acetylglucosamine. Arch Biochem Biophys.

